# Indents

**Published:** 1912-01-15

**Authors:** 


### INDENTS.

☞Brief Articles of Technical or Scientific Value will receive notice and comment. Contributions invited.

*A Hope for the Elderly.* Dr. Hard, a writer in the *Medical Times*, presents us with a new proposition for the staving off of old age. His remedy is is a new one, though the proposition is many centuries old. The ancient alchemists devoted many years of investigation to to a search for the Philosopher's Stone and the Elixir of Life. Later, Jaun Ponce De Leon searched the Bahamas and the coast of Florida for the Fountain of Youth. A quarter of a century ago, dilute phosphoric acid was the remedy, because it was believed that its consumption prevented calcification of tissue. Now, it is suggested that thiosinamine is the proper agent to soften hardened tissue and prevent its aging.

Thiosinamine is a crystalline deposit which occurs when one part of volatile oil of mustard is mixed with one part of alcohol and two parts of ammonia water and allowed to stand for a few hours, after being well shaken. It is also found in horse-radish root. We have been unable to find any account of its therapeutic property except the statement of Dr. Hard, that "Thiosinamine has proven to act as a resolvent of indurated flesh."

He writes: Whether it acts by changing back into normal cells those which have stepped down from active to simple connective cells, we do not know. It may be that it stimulates the deposit of original cells in increasing numbers where the supply was short; or it may increase the natural life energy of existing cells, preventing degeneracy in those which otherwise would succumb to debilitating influences; or it may act indirectly, in decreasing the burden of duty devolving upon the already located cells. However it may act, we are justified in its use for the purpose indicated, and I

suggest that thiosinamine be given in one-tenth grain doses three times a day, for the purpose of staying the hand of Time, and keeping a man from 'growing old.' "

Dr. Hard's object is certainly praiseworthy, and many of us would undoubtedly rise up and call him blessed if his suggestion would even hold us where we are. We have seen the time, when people lacked faith in us on account of our youthful appearance, that thiosinamine would have had no charms; but, if it will do what it is expected of it in the Doctor's mind, it certainly looks good to us now.—Dr. Webster, in *Electic Medical Journal.*

*Compressed Oxygen.* REPORT OF THE COUNCIL ON PHARMACY AND CHEMISTRY.

The referee of the Council appointed to report on the composition of compressed oxygen as found on the market and to make recommendations as to its inclusion with New and Nonofficial Remedies submitted the following report:

The Section on Stomatology at the 1909 session of the American Medical Association having recommended the inclusion of compressed oxygen with the U. S. Pharmacopeia, the council voted to describe this product with New and Non official Remedies until such Pharmacopeial recognition had been secured. This action having been decided on, it became necessary to provide standards of purity for this substance. Correspondence both with firms who make the product as well as with those who use it gave the referee of the Council little information as to what degree of purity should be required for this substance, and it therefore became necessary to examine the product as it was found on the market.

Professor Warren R. Smith of Lewis Institute, Chicago, kindly offered to cooperate with the Council and to make an examination of this product. The referee herewith transmits the very thorough report regarding the composition of commercial compressed oxygen which has been made by Professor Smith in collaboration with Edwin D. Leman. The referee recommends that the Council express its appreciation for the work and that it request publication of the report in THE JOURNAL. The referee further recommends that the description of compressed oxygen submitted herewith be adopted for inclusion with New and Nonofficial Remedies.

This report was adopted by the Council and in accordance therewith the examination of Smith and Leman is published below and the description of compressed oxygen appears with New and Nonofficial Remedies on another page of this issue of *The Journal.*

W. A. PUCKNER, *Secretary.*

### The Purity of Compressed Oxygen.

PROF. WARREN R. SMITH AND EDWIN D. LEMAN.

We have recently examined three specimens of commercial oxygen, purchased in the open market, with the results shown in the accompanying table.

| Maker | Chicago Oxygen Gas Co. Chicago | Ohio Chemical and Mfg. Co. Cleveland | S. S. White Dental Mfg. Co. Philadelphia |
|---|---|---|---|
| Per cent. oxygen | 94.78 94.80* / 94.78 94.80† | 97.84 97.85* / 97.84 97.86† | 96.84 96.76* / 96.84 96.93† |
| Per cent. nitrogen[1] | 5.22 5.20* / 5.22 5.20† | 2.15 2.16* / 2.14 2.16† | 3.09 3.17* / 3.09 3.00† |
| Potassium iodid test | Negative. | Negative. | Negative. |
| Per cent. carbon dioxid | Negative. | Negative. | 0.06 0.07* |
| Carbon monoxid methane, etc | Negative. | Negative. | Negative. |

1. That is, not absorbed by potassium pyrogallate.

*Smith.

†Leman.

The Gas from the Chicago Oxygen Co. was claimed to be under a pressure of 16 atmospheres. The amount of nitrogen found in this sample is just about that which would be present if pure oxygen were forced into a container filled with air under one atmosphere pressure until a pressure of 16 atmospheres was reached. The other samples were stated to be under a pressure of 1,600 lbs., so that the amounts of nitrogen found cannot be accounted for by a similar hypothesis.

The potassium iodid test mentioned in the table consisted in passing from 2 to 4 liters of the gas through a solution containing starch and potassium iodid. A negative result in this test shows that the gas is free from chlorin, ozone, oxids of nitrogen, etc. The negative results for carbon dioxid mean that not more than a slight opalescence was obtained by passing 2 to 4 liters of the gas through a solution of barium hydroxid. The carbon monoxid test was made by passing the gas through alkali to remove all carbon dioxid, then over heated platinum, and finally through barium hydroxid solution. A negative result here shows freedom from carbon monoxid, methane and other hydrocarbons.—*Journal A. A. M.*

*Enlarge Opening When Filling Pulp Canals.* A very common mistake is made in trying to fill pulp canals in bicuspids and molars through too small an opening, which is almost sure to result, in many cases, in imperfect root canal fillings. It is far better to sacrifice the crown of the tooth or some portion of it than it is to take chances of losing the whole tooth. Well filled root canals means the saving of the foundation. The crown can always be restored either with a filling, inlay or an artificial crown.—J. Austin Dunn.—*Review.*

*Varnish for Models.* White wax dissolved in spirits of turpentine makes an excellent varnish for models. Orthodontia models, etc., not intended for use on the workbench are more pleasing and artistic varnished with a transparent spirit varnish.—*Brief.*

*To Mend Broken Plaster.* Have a jar of sandarach about as thick as cream. Coat the two parts, then hold them in the Bunsen flame and while the alcohol is burning clamp them together. This is the only method I have ever found that is absolutely certain.—G. E. Hawkins, *Review.*

*Controlling Hemorrhage in Setting Crowns and Bridges.* In irritated conditions where the gums have a tendency to weep and bleed, treat the gum margin with a fifteen percent solution of trichloracetic acid. I know of no other astringent that so absolutely controls such a condition in setting crowns and bridges and inlays, and at the same time having such a curative effect.—J. E. Argue, Red Lake Falls, Minn., *Dental Review.*

### DENTIFRICE.

The Philadelphia Free Dental Clinic for School Children has adopted the following formula:

| | |
|---|---|
| Precipitated chalk | 95 |
| Castile soap | 3 |
| Saccharin | $\frac{1}{8}$ |
| Oil of birch | 1 |
| Oil of peppermint | $\frac{1}{2}$ |

This has been manufactured in quantity for free distribution, each package labeled with the formula, and with directions for its use and the proper method of using a tooth brush.—*Brief.*

*Taking Partial Impressions with Wax and Plaster.* Dr. S. G. Watkins, in the *Brief* makes this suggestion. Take the impression in beeswax, removing it before it becomes at all hard. Then, by molding with his fingers or cutting away with a knife, he makes room for a liberal thickness of plaster when retaking the impression, using the wax impression as an impression tray. Before doing so, however, he carefully molds to the remaining natural teeth a single thickness of base-plate wax, well softened, so that it may be forced well between and closely to the teeth. Then, placing sufficient plaster in the wax impression, he forces it firmly to place in the mouth, keeping up the pressure until the plaster has hardened. The base-plate wax comes away with the impression, forming part of it, and gives a sharper impression of the teeth than is usually obtained with plaster alone, and, furthermore, its plasticity favors the removal of the impression without undue force.

*Post for Crown.* Here is a little practical hint that is a good one and a money saver. We have all had our failures from corrosion by using anything other than iridio-platinum for posts, and on account of the price are tempted at times to use something else.

You can use any of these substitutes by first fluxing the post and wrapping a little gold foil around same and applying the flame and flowing a small piece of 22 K. solder which the gold takes up. This only takes a moment and is a great saving.—H. L. Entriken, Enid, in *Western Journal.*

*Remedy for Burns.* Dentists, as a rule, have at hand one of the most efficient and soothing remedies for burns, viz., grain alcohol. Saturate cotton or lint, cover burn with same and keep moist until pain subsides, which will be in a very short time. Healing is by first intention in nearly every case, regardless of the depth or extent of the burn. Alcohol also neutralizes the effect of carbolic acid on mucous membrane.—E. G. McAbery, in *Review.*

*Turpentine as a Lubricant.* Where oil will not act as a cooling agent on a drill working on hard metals, turpentine used instead will permit the drill to take hold and retain its temper.—*Brief.*

*To Avoid Excess of Water in Investments for cast Inlays.* We read much about accuracy in mixing investments for cast inlays. Instruments are on the market for weighing and measuring the water and investment used, but it is impossible to get a mix that will pour without an excess of water. Weigh a fresh plaster cast or investment cast of combined materials, dry, and weigh again—the difference is the excess of water not required to satisfy crystallization in the plaster. Some excess is of course necessary in any method. Now how shall we reduce the water to the minimum?

First select a flask deep enough to allow the wax model to be well below the top of the flask, and sift the investment into the water, pouring off the excess. Take care of your model with a camel's hair brush, pour the investment until the model is well covered, and the investment within about one quarter of an inch of the top, and sift in dry investment until it rises in a cone above the flask. Then wait a few minutes until the water rises from the saturated investment below, and press the cone gently down; repeat the process if necessary, using care and judgment in the pressure and amount of dry material used, and you will soon learn to make an almost perfect investment so far as the right proportion of water is concerned. Don't give up if you happen to make one too dry and rob the lower portion of water.

By this method you can place your flask safely on a pretty hot surface in five minutes, and in ten minutes transfer it to the gas flame.—Dr. George E. Smith, in *Cosmos.*

